# Enteral versus intravenous antibiotics for critically ill patients: A pilot study

**DOI:** 10.1016/j.bjid.2025.104538

**Published:** 2025-05-14

**Authors:** Dayana dos Santos Oliveira, Julia Vitória da Rocha, Juliano Gasparetto, Carolina Hikari Yamada, Joao Paulo Telles, Felipe Francisco Tuon

**Affiliations:** a Pontifícia Universidade Católica do Paraná (PUCPR), Hospital Universitário Cajuru, Curitiba, PR, Brazil; b Pontifícia Universidade Católica do Paraná (PUCPR), Faculdade de Farmácia, Curitiba PR, Brazil; c Pontifícia Universidade Católica do Paraná (PUCPR), Laboratório de Doenças Infecciosas Emergentes, Curitiba PR, Brazil; d Hospital Universitário Evangélico Mackenzie, Departamento de Doenças Infecciosas, Curitiba, PR, Brazil

**Keywords:** Antimicrobial stewardship, Enteral antibiotic, Cost-minimization, Oral switch, Intensive care unit

## Abstract

**Background:**

While there are valid concerns regarding the use of Enteral Antibiotics (EN) in the Intensive Care Unit (ICU), the evidence opposing this practice has not been thoroughly evaluated in prior research.

**Aim:**

This study aimed to evaluate the transition from sequential Intravenous (IV) antibiotics to EN antibiotics in critically ill patients with infections.

**Methods:**

This was a prospective, multicenter, randomized, unblinded clinical trial involving patients with infections who received antibiotic therapy. The study compared sequential IV antibiotic treatment to EN therapy in patients who continued standard IV therapy in the intensive care unit. Primary outcome measures included mortality and clinical improvement assessed on days 3, 5, and 10. Secondary outcomes encompassed hospital and ICU length of stay, costs, and evaluation of microbiological failure.

**Results:**

A total of67 patients were included in the EN group and 60 patients in the IV group. Most patients were classified as infected (66.1 %), with 33.1 % diagnosed with sepsis. In-hospital mortality rates were comparable between the two groups, with 31 % in the EN group and 30 % in the IV group. Clinical outcomes assessed on days 3, 5, and 10 showed no significant differences between the groups. Among the 67 patients in the EN group, 7 (10.5 %) required a return to intravenous antibiotic therapy. Notably, drug costs in the IV group increased by 207 %.

**Conclusion:**

This is the first controlled and randomized study to evaluate the oral/enteral route of antibiotic administration in the ICU. The findings indicate no significant differences in clinical outcomes or survival rates between the two groups, while demonstrating reduced costs and comparable safety with EN antibiotics.

## Highlight

• In this randomized study of sequential therapy, intravenous and enteral antimicrobial therapy was similar in survival and clinical improvement.
• In hospitalized patients, after an initial clinical stabilization, 83 % of patients could be using intravenous antibiotics unnecessarily.
• Drug costs in the intravenous antibiotics increased by 207 % when compared with enteral antibiotics.

## Introduction

Most patients in the Intensive Care Unit (ICU) receive Intravenous (IV) antibiotics due to several factors, including the severity of their infections and the low bioavailability of certain Enteral (EN) antibiotics. Additionally, despite some antibiotics exhibiting high bioavailability, concerns remain regarding gastrointestinal function in the early stages of sepsis^1^, ^2^, ^3^ Notably, after an initial stabilization period of72 h, it is estimated that 83 % of hospitalized patients are using IV antibiotics unnecessarily, leading to a 200 % increase in costs for some antibiotic classes and prolonging hospital stays^4^

While the literature supports the transition from IV to EN antibiotics in hospitalized patients, data on this approach for critically ill patients are limited^5^ Although there are concerns regarding the use of EN antibiotics in the ICU, some, like levofloxacin, demonstrate bioavailability comparable to IV administration, even in critically ill patients^6^ Some intensivists believe that vasoactive medications may impair enteral absorption; however, these drugs have minimal impact on mesenteric blood flow^7^ Switching from IV therapy to the oral route offers several potential benefits, including early discharge, a reduced risk of bacteremia, decreased reliance on venous access and the associated risk of thrombophlebitis, and lower treatment costs^8^^,^^9^ An emerging concern in healthcare is the sustainability agenda, where the enteral route significantly reduces plastic and disposable waste compared to the parenteral route, adding another compelling reason to consider this shift. Furthermore, the financial implications of oral antibiotics are particularly significant in developing countries. However, several studies on antibiotic consumption in Brazilian hospitals do not support the use of oral antibiotics^10^

Although there is evidence demonstrating the effectiveness of EN antibiotics in ICU patients, there are currently no controlled and randomized clinical studies confirming the non-inferiority of this approach. This study aimed to evaluate the transition from IV to EN antibiotic administration in critically ill patients.

## Methods

### Study design

This was a prospective, multicenter, randomized, unblinded clinical trial conducted in the Intensive Care Unit (ICU) involving patients with infections receiving antibiotic therapy. The blinding of the teams in this study would be challenging due to the variety of medications used in the treatment, as well as the prescription modifications based on the individualized characteristics of each patient. The study compared sequential IV therapy to EN therapy, with one group (EN group) transitioning to EN antibiotics, while the other group (IV group) maintained standard IV therapy. The term “enteral” encompasses oral administration, as well as orogastric, nasogastric, and naso-enteric tubes. We considered a non-inferiority study with a sample power of 70 %, confidence interval of 90 %, and non-inferiority limit of 15 % for calculation the study population. In this study, 60 patients were considered in each group (Group 1 and 2) and randomized in a 1:1 ratio of IV and EN antibiotics. The percentage 'success' in control group would be 80 % (survival). See https://www.sealedenvelope.com/power/binary-noninferior/.

### Study setting

Patient enrollment took place at two Brazilian hospitals, both specializing in trauma, neurosurgery, general surgery, and medical patients. A total of 120 ICU beds were included in this study. The local ethics committee approved the study prior to randomization, which occurred from April 23, 2020, to April 2022 (ethical committee approval n° 3.987.566). Informed consent was obtained from conscious patients or their legal representatives in the case of unconscious patients.

### Sample size

The sample size was determined based on convenience, with 60 patients allocated to each group (EN and IV) using a 1:1 randomization ratio.

### Study participants

Inclusion criteria

1) Age > 18-years;
2) Admission to the ICU;
3) Clinical diagnosis of infection;
4) Regular oral or enteral feeding;
5) Availability of enteral antibiotics with equipotency to IV alternatives;
6) Signed consent from the patient or ICU team;
7) At least 24-hours of clinical improvement.

Exclusion Criteria:

1) Life expectancy < 24-hours;
2) Treatment considered futile;
3) Diet intolerance or refusal of EN medications;
4) High gastrointestinal bleeding;
5) Lack of suitable EN antibiotic alternatives due to microbiological results;
6) Patients with COVID-19 were also excluded.

### Recruitment and randomization

After fulfilling the inclusion criteria, patients were randomized using an online randomization system (www.random.org). A clinical pharmacist oversaw the randomization process. In the EN group, patients transitioned from IV to EN antibiotics as determined by the attending physician in collaboration with the antimicrobial stewardship team (including a clinical pharmacist and an infectious disease specialist). Patients in the IV group continued their IV therapy. The researcher did not influence the attending physician's decisions regarding the duration of therapy. Supplementary Tables 1 and 2 detail the commercially available options for EN use and the method of administration (oral or via EN tube), respectively. The antimicrobial stewardship team was responsible for screening and assessing patients.

The decision to switch back to intravenous therapy was based on well-established clinical criteria to ensure it is justified and avoids unnecessary biases. Some key aspects were considered: i) True therapeutic failure: The need to return to IV antibiotics confirmed by objective signs, such as the persistence or worsening of symptoms, prolonged fever, increased inflammatory markers (CRP, ESR, procalcitonin), and positive microbiological cultures indicating treatment failure; ii) Conservative medical decision: In some cases, physicians have opt for an early return to IV therapy due to excessive precautions, even without clear evidence of failure.

### Clinical and microbiological data

Clinical and laboratory data collected included sex, age, total duration of IV and EN antibiotic therapy, duration of mechanical ventilation, length of hospital and ICU stay, use of vasoactive drugs, comorbidities (Charlson index), and clinical outcomes. The Acute Physiology and Chronic Health Disease Classification System II (APACHE II) and Sequential Organ Failure Assessment (SOFA) scores were assessed on the day of randomization. All identified microorganisms and infection sites were evaluated, and antimicrobial adequacy was assessed according to susceptibility tests. Empirical antibiotics with negative culture results were excluded from this subgroup analysis.

### Severity of infection

The severity of infection was classified using the following sepsis criteria:

• Sepsis ‒ Organ dysfunction resulting from a dysregulated host response to infection, indicated by an acute increase of ≥ 2 points in SOFA scores due to infection.
• Septic Shock ‒ Hypotension requiring vasopressors to maintain a mean arterial pressure > 65 mmHg and a serum lactate level > 2 mmoL/L despite adequate fluid resuscitation^11^

Infection definitions adhered to CDC criteria, as all patients presented with healthcare-associated infections^12^

### Study outcomes

The primary outcome was patient mortality, with in-hospital and 30-day mortality and survival curves evaluated. Secondary outcomes included clinical response, while tertiary outcomes encompassed the length of hospital and ICU stay. Microbiological failure was assessed, defined as the persistence of the same microorganism at the infection site. Patients requiring a return to IV therapy due to treatment failure were classified as clinical failures. The cost analysis for each group included the costs of the drugs, syringes, needles, diluents, and carrier fluids.

Definition of clinical improvement

Each infection type has specific signs and symptoms for evaluation. Therefore, general criteria for clinical improvement of sepsis were considered on days 3, 5, and 10^13^ Patients demonstrating at least 24 h of clinical improvement or stability were defined by criteria including temperature < 37.8 °C, heart rate < 100 beats/min, respiratory rate < 24 breaths/min, systolic blood pressure > 90 mmHg, arterial oxygen saturation > 90 % or pO_2_ > 60 mmHg in room air, the ability to maintain EN intake, and a normal mental status (according to the Glasgow scale)^14^

### Statistical analysis

Descriptive data were reported as percentages, with quantitative data presented as means or medians, depending on the distribution pattern. Standard deviations and interquartile ranges (25th and 75th percentiles) were used for mean and median distributions, respectively. Associations between variables and outcomes were analyzed using the Student's *t*-test, Mann-Whitney test, Chi-Square test, or Fisher's exact test, with a p-value < 0.05 considered statistically significant. For multivariate analysis, all variables showing significance in univariate analysis were included in binary logistic regression. Survival curves (Kaplan-Meier) were constructed from the initiation of antibiotic therapy until patient death or discharge. Overall and 30-day mortality curves were generated, and the Gehan-Breslow-Wilcoxon test was conducted. All analyses were performed using SPSS version 23.

## Results

### Patient characteristics and presentation

**Fig. 1:**
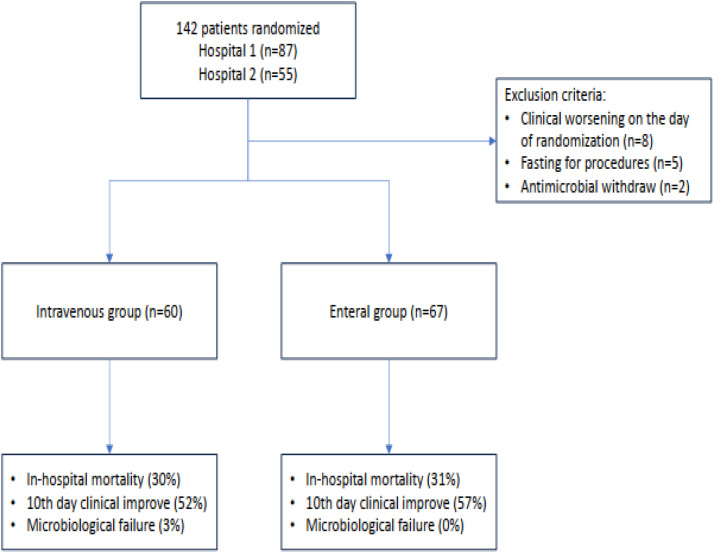
Flowchart of patients randomized to maintain IV antibiotic or switch to enteral route admitted into the ICU with a diagnosis of infection.

Of the 142 eligible patients, 15 were excluded prior to randomization. The primary reasons for exclusion included: (i) Clinical deterioration on the day of randomization (53 %); (ii) Fasting for examinations (33 %); and (iii) Withdrawal of antimicrobial therapy on the day of randomization (13 %) (Fig. 1). After exclusions, 67 patients were included in the EN and 60 in the IV group. Baseline patient characteristics at the time of randomization are summarized in Table 1. The groups were comparable in terms of age, sex, total length of hospital and ICU stay, comorbidities, and severity scores (SOFA and APACHE II).

**Table 1:** Baseline data of patients randomized to maintain IV antibiotic (group IV) or switch to enteral route (group EN) and outcomes.

	Group EN		Group IV		p-value
	*n* = 67	%	*n* = 60)	%	
	n or (mean ± SD)		n or (mean ± SD)		
**Baseline characteristics**					
Gender (male)	52	78 %	39	65 %	0.084
Age (mean ± SD)	56.8 ± 18.8		55.1 ± 0.9		0.768
HIV infection	2	3 %	0	0 %	0.276
Diabetes mellitus	12	18 %	12	20 %	0.469
Chronic renal failure	1	1 %	2	3 %	0.452
Chronic heart failure	2	3 %	1	2 %	0.547
Dementia	1	1 %	1	2 %	0.723
Chronic lung disease	8	12 %	8	13 %	0.511
Neoplasm	2	3 %	2	3 %	0.647
Cirrhosis	0	0 %	1	2 %	0.276
Charlson index	0.8 ± 0.9		1.1 ± 1.1		0.204
ICU lengh of stay before randomization	6.7 ± 8.1		6.4 ± 6.7		0.797
SOFA score	4.1 ± 3.4		5.2 ± 3.5		0.101
APACHE II	15.5 ± 8.3		18.1 ± 7.8		0.088
Mechanical ventilation	43	64 %	40	67 %	0.457
Enteral nutrition	45	67 %	47	78 %	0.113
Oral	22	33 %	13	22 %	0.113
Vasoative drug	18	27 %	16	27 %	0.570
Creatinine (mg/dL)	1.2 ± 1.7		1.4 ± 1.5		0.540
**Infection and antibiotic History**					
Site of Infection					0.557
Bloodstream infection	2	3 %	4	7 %	
Respiratory	53	79 %	45	75 %	
Soft tissue	3	4 %	6	10 %	
Urinary	6	9 %	2	3 %	
Abdominal	2	3 %	2	3 %	
Other	1	1 %	1	2 %	
Classification of infection					0.385
Sepsis	20	30 %	22	37 %	
Septic shock	0	0 %	1	2 %	
Infection	47	70 %	37	62 %	
Antibiotic therapy					
Empirical	32	48 %	19	32 %	0.047
Culture guided	35	52 %	41	68 %	
Days of antibiotic before randomization	2.6 ± 1.4		2.9 ± 1.7		0.239
Duration of antibiotics after randomization	4.2 ± 3.4		4.7 ± 4.1		0.367
Outcomes					
Lengh-of-stay after randomization (days)	34.1 ± 34.6		36.9 ± 37.9		0.663
Duration ICU Admission	18.8 ± 18.3		13.4 ± 1.6		0.060
In-hospital Mortality	21	31 %	18	30 %	0.512
3rd day clinical outcome					
Improve	56	84 %	45	75 %	0.485
Failure	2	3 %	3	5 %	
Indifferent	9	13 %	12	20 %	
5th day clinical outcome					
Improve	46	69 %	40	67 %	0.969
Failure	3	4 %	3	5 %	
Indifferent	18	27 %	17	28 %	
10th day clinical outcome					
Improve	38	57 %	31	52 %	0.795
Failure	5	7 %	4	7 %	
Indifferent	24	36 %	25	42 %	
Microbiological failure	0	0 %	2	3 %	0.285

ICU, Intensive Care Unit.

The most common site of infection was the respiratory tract (77.1 %), followed by soft tissues (7.0 %). Based on sepsis criteria, the majority of patients were classified as having an infection (66.1 %), followed by sepsis (33.1 %), with only one patient meeting the criteria for septic shock (0.8 %). Approximately 25 % of all patients were receiving vasoactive drugs at baseline, though only one patient fulfilled the criteria for septic shock. There were no significant differences in sepsis classification between the IV and EN groups (*p* = 0.385).

The predominant pathogens identified were Gram-positive cocci, primarily *Staphylococcus aureus* (49 %), followed by *Enterobacterales* (34 %), with *Klebsiella* spp. (12 %) and *Escherichia coli* (10 %) being the most common. Only 13 % of infections were polymicrobial (Supplementary Tables). Culture results by infection site are detailed in Supplementary Table 3.

The sole difference observed between the IV and EN groups was in the type of antibiotic therapy received, with microbiologically guided treatment more frequently employed in the IV group compared to the EN group (68 % vs. 52 %, *p* = 0.047). Empirical therapy was utilized in 48 % of the EN group and 32 % of the IV group. The duration of antibiotic use was similar between both groups.

Before randomization, the most commonly used antibiotics were quinolones (levofloxacin or ciprofloxacin) at 37 %, followed by ceftriaxone at 21 % (Table 2). Monotherapy was more prevalent, particularly in cases of respiratory tract infections. Following randomization, quinolones remained the most prescribed antibiotics in the EN group (47 %), followed by amoxicillin with clavulanate (24 %) and sulfamethoxazole-trimethoprim (18 %). Among patients with microbiological identification of their infections, treatment was deemed adequate in 86 % (65/76) prior to randomization.

**Table 2:** Antibiotics used before and after randomization of the patient to maintain IV therapy or switch to enteral therapy.

	Before randomization		After randomization (Only for enteral therapy)	
Antimicrobial therapy	n	%	n	%
Monotherapy				
Intravenous				
Levofloxacin/ciprofloxacin	47	37 %		
Ceftriaxone	27	21 %		
Vancomycin	16	13 %		
Aminoglycoside	15	12 %		
Sulfamethoxazole/trimethoprim	8	6 %		
Ceftazidime	1	1 %		
Oral/enteral				
Levofloxacin/ciprofloxacin	‒	‒	31	47 %
Amoxicillin/clavulanate	‒	‒	16	24 %
Sulfamethoxazole/trimethoprim	‒	‒	12	18 %
Doxicycline	‒	‒	4	6 %
Combination therapy				
Intravenous				
Cefriaxone + (clindamycin/metronidazole)	5	4 %		
Aminoglycoside + Sulfamethoxazole/trimethoprim	2	2 %		
Aminoglycoside + levofloxacin/ciprofloxacin	2	2 %		
Aminoglycoside + (clindamycin/metronidazole)	1	1 %		
Cefepime + vancomycin	2	2 %		
Meropenem + vancomycin	1	1 %		
Oral/enteral				
Levofloxacin/ciprofloxacin + Sulfamethoxazole/trimethoprim			1	2 %
Levofloxacin/ciprofloxacin + metronidazole			2	3 %

### Outcome

**Fig. 2:**
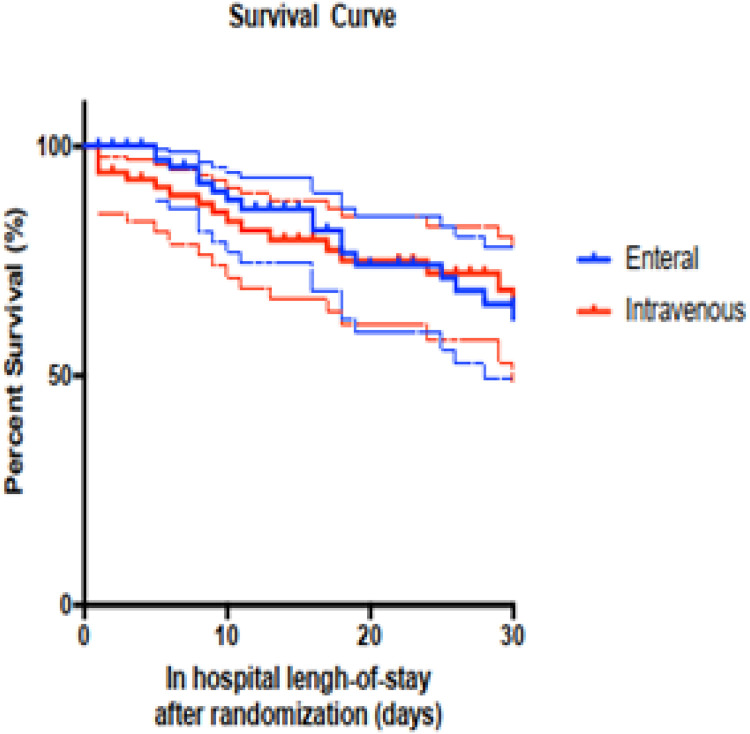
Survival curve of patients randomized to maintain IV antibiotic or switch to enteral route admitted into the ICU with a diagnosis of infection. Dashed line represents the Hazard Ratio (HR) with a 95 % Confidence Interval.

The in-hospital mortality rates were comparable between the two groups, with 31 % in the EN group and 30 % in the IV group. Additionally, clinical outcomes assessed on days 3, 5, and 10 ‒ categorized as improvement, failure, or indifferent ‒ showed no significant differences between the groups. The 30-day survival Kaplan-Meier curve is presented in Fig. 2, illustrating similar survival rates for both the IV and EN groups (*p* = 0.992).

In the IV group, only two patients experienced microbiological failure. Among the 67 patients in the EN group, 7 (10.5 %) required a return to the IV route of antibiotics. The reasons for this transition included two treatment failures without microbiological identification, two cases where bacteria were susceptible only to IV antibiotics, and three patients who experienced gastric stasis. Those patients who needed to revert to IV therapy due to treatment failure were classified as clinical failures.

### Costs impact

The total cost of antibiotics in the EN group was USD 1243.00, which included pre-randomization IV antibiotics. The total cost of IV group was USD 2575.35, with an increase of 107 %.

## Discussion

The administration of antibiotics via the enteral route raises concerns among the Intensive Care Unit (ICU) healthcare team due to potential issues related to suboptimal drug absorption and reduced bioavailability. These factors may jeopardize the achievement of crucial Pharmacokinetic and Pharmacodynamic (PK/PD) targets necessary for therapeutic efficacy. Our investigation found that using enteral antibiotics in patients with infections, including those with sepsis, did not result in significantly different clinical outcomes compared to Intravenous (IV) antibiotics. Importantly, this approach also led to cost savings, although two patients experienced therapeutic inadequacies while receiving enteral therapy.

Cunha and colleagues have proposed that in instances of substantial bioavailability, a reassessment of the potential transition to the oral route should be considered.^15^ Certainly, this approach may not be advisable during the initial hours of treatment, especially when the patient is in a heightened inflammatory state. It's important to recognize that in these early stages, sepsis-induced gastroparesis can hinder the absorption process, potentially leading to therapeutic failure.^15^ When utilizing medications with high bioavailability, such as quinolones, metronidazole, clindamycin, doxycycline, and sulfa drugs, it is highly likely that the achievement of pharmacokinetic and PK/PD targets is ensured.^15^, ^16^, ^17^ The achievement of the pharmacokinetic and PKPD target is contingent upon the Minimum Inhibitory Concentration (MIC) of the relevant microorganism. It is imperative that antimicrobial stewardship takes cognizance of these values to guarantee a favorable clinical response. Consequently, our findings align with those presented by Cunha and colleagues, who have proposed that the IV route remains pivotal, particularly for patients undergoing initial therapy, in cases where gastrointestinal absorption is suboptimal, or when oral alternatives do not align with microbiological findings.

When considering enteral therapy, it is essential to account not only for achieving drug concentrations that meet PK/PD targets but also for the susceptibility profile of the pathogens involved. Notably, nearly 50 % of the enteral therapy group in our study received empirical treatment. Our investigation found that even among ICU patients, the prevalence of multidrug-resistant bacteria was relatively low, leading to clinical responses comparable to those seen with intravenous therapy; however, this is a pilot study. However, in healthcare settings with a high prevalence of multidrug-resistant bacteria, we recommend that enteral therapy be guided only after obtaining susceptibility test results. In such cases, utilizing a cumulative antibiogram from the antimicrobial stewardship team would be an optimal strategy^18^ There is a myth that vasoactive drugs can impairs the enteral absorption; however, norepinephrine (the major vasoactive drug used in our study) has minimal impact on mesenteric blood flow^7^

The cost reduction of sequential IV to EN therapy is obvious, considering the cost of the oral drugs and supplies used for infusion. Some studies have demonstrated these results in common infections, such as community-acquired pneumonia,^19^ urinary tract infections,^20^ intra-abdominal infections,^21^ as well as osteomyelitis^22^ We previously published a retrospective study of EN therapy in the ICU, comparing this strategy with patients who received IV therapy^9^ The impact was significant in terms of costs and length of hospital stay, without any impact on the outcome. However, the limitation of that study was its retrospective design. Thus, we designed the current study to confirm the results, including randomization and continuous follow-up of the patients. The IV route increases the cost of antibiotics by >200 %.

Antimicrobial resistance is commonly observed in ICUs^23^ These data highlight the need for ASP teams to reduce antimicrobial resistance, while still in time. Nevertheless, if traditional options, such as carbapenems for Extended Spectrum Beta-Lactamases (ESBL) isolates and vancomycin for Methicillin Resistant *S. aureus* (MRSA) isolates continue to be used to treat them, there will be few strategies that can significantly impact resistance. Even in the presence of resistance, oral antimicrobial options can be made, such as doxycycline for *Acinetobacter* spp., quinolones for ESBL or ampC, and sulfamethoxazole-trimethoprim for MRSA^18^ Therefore, once a patient achieves minimum clinical safety to oral switching, a change in therapy can be performed. However, clinicians should be aware that the criteria can be categorically defined and do not rely only on general perception. Based on the same ASP protocol, our team demonstrated important hospital costs and resistance impacts.^24^

Recent studies have demonstrated that “alternative” options may reach similar outcomes like “traditional” options. For instance, in a case-control study, sulfamethoxazole-trimethoprim was found to be similar to vancomycin^25^ Non-carbapenem options (e.g., quinolones) to treat ESBL tract infection have also been demonstrated to be non-inferior to carbapenem options^26^ Nevertheless, despite these studies not evaluating only oral-based therapy, antimicrobial oral switch could be used, and it has been an issue of review more than 20-years ago^27^ Moreover, we recall that the literature provides strong evidence for the use of oral antibiotics in patients outside the ICU, including those who are hospitalized, such as for pneumonia^28^; abdominal infection,^29^ even for meningitis^30^^,^^31^ In critical patients, other studies have shown the safety of this strategy^32^^,^^33^

This study was designed to reduce bias in our previous retrospective study^9^ considering a pilot study, despite of randomization, selection bias was possible, considering that empirical therapy was more frequent in the EN group, a possible benefit due to 1) Non-infectious disease; and 2) Low bioburden to recover the microorganism in classical microbiology (no molecular test was used). The primary endpoint selected for our study was mortality. Nevertheless, evaluating differences in mortality among critically ill patients is a complex endeavor. Attributing death to a specific treatment modality in this population proves challenging due to the substantial number of adverse events, the considerable variability in clinical conditions, and the presence of diverse complications^34^ This may include time on mechanical ventilation attributed to age, lung diseases, as well as muscle changes that lead to reduced mobility, risk of deep vein thrombosis, bronchoaspiration, among others^35^, ^36^, ^37^ As an open label study in which exclusion criteria included at the “refusal of the care team”, patients who were “sicker” were denied consideration out of fear that they would not do well with enteral antibiotic therapy. There are some confounders for data clinical improvement – e.g., vasopressor changes could affect heart rate, and opioid use could affect respiratory rate independent of effect of antibiotics.

Withdrawal of patients after randomization to enteral arm, and reversion back to IV route needs attention, because is a confounding bias, considering that reversion to IV route is considered a failure of enteral route, but also a change due to microbiological result with only IV alternatives.

In conclusion, the EN route of antibiotic administration in the ICU can lead to cost reductions while achieving similar clinical outcomes. However, further studies involving larger patient populations are needed to confirm the safety and efficacy of this approach. It is important to consider this strategy in patients with clinical stability in the last 24 to 48 h, a functional digestive tract, high-bioavailability drugs, and always under the supervision of an antimicrobial stewardship team to provide advanced support in pharmacotherapeutic management.

## Conflicts of interest

The authors declare no conflicts of interest.

## Appendix Supplementary materials

Image, application 1
