# Supplementary material for: Enteral versus intravenous antibiotics for critically ill patients: A pilot study
Source: Braz J Infect Dis. 2025 May 14;29(4):104538. doi: 10.1016/j.bjid.2025.104538 (PMC12144447; doi:10.1016/j.bjid.2025.104538)
Supplement: Supplementary file 1 [file mmc1.docx]

**BJID-D-24-00354_ Supplement material**

**Supplement material**

**Table S1** Antibiotics subject to pathway descaling.

| **Drug** | **Intravenous presentation** | **Enteral presentation** | **Enteral Bioavailability** |
| --- | --- | --- | --- |
| Amoxicillinᵃ |  | 500 mg | 60%‒90% |
| Amoxicillin / Clavulanic acid | 1g / 200 mg | 500 / 125 mg | 60%‒90% |
| Azithromycinᵃᵇ | 500 mg | 500 mg | 60%‒90% |
| Cephalexin |  | 500 mg and 1g | >90% |
| Cefuroxime | 750 mg | 250 and 500 mg | >90% |
| Ciprofloxacinᵇ | 200 and 400 mg | 500 mg | >90% |
| Clindamycin | 600 mg | 300 mg | >90% |
| Clarithromycin |  | 500 mg | 50% |
| Doxycycline |  | 100 mg | 60%‒90% |
| Fluconazoleᵇ | 200 mg | 150 mg | >90% |
| Levofloxacinᵃ | 500 mg | 500 mg | 99% |
| Linezolid | 600 mg | 600 mg | 100% |
| Metronidazoleᵇ | 500 mg | 400 mg | 100% |
| Moxifloxacin | 400 mg | 400 mg | >90% |
| Nitrofurantoinᵃ |  | 100 mg | 87% fasting/ 94% with food |
| Rifampicinᵇ |  | 300 mg | >90% |
| Sulfamethoxazole / Trimethoprim | 400 / 80mg / 5mL | 400 / 80 mg | 90%‒100% |

^a^ Micromedex^®^ Healthcare Series [Internet database]. Greenwood Village, Colo: Thomson Healthcare.

^b^ Cyriac JM, James E. Switch over from intravenous to oral therapy: A concise overview. Journal of pharmacology & pharmacotherapeutics. 2014 Apr; 5(2):83.

**Table S2** Antibiotic drugs that can be administered by tube Administration by tube: The ideal formulation for tube passage is solution or suspension, but not all drugs are available in this presentation. When the only option is the tablet, it is necessary to consult the attached table. If the chosen drug is not in the table, the administration of this tube is not indicated. Dilution must be performed with distilled water in the syringe, wait for complete dissolution, and administer via tube. When the formulation is a capsule, it must be opened, and its contents mixed with distilled water in the syringe and administered via a tube. Administer 20 mL of distilled water before and after each medication to avoid obstructions and ensure full dose administration. Stop feeding before administration, according to the particularity of each drug described in the Table 2.

| **Drug** | **Orientation** |
| --- | --- |
| Amoxicillin (500 mg caps) | It must not be broken or crushed. Open the capsule and disperse in 10 mL of water. |
| Amoxicillin + clavulanic acid (500 + 125 mg, coated tablet) | It must not be broken or crushed. Open the capsule and disperse in 10 mL of water. |
| Azithromycin (500 mg, tablet) | You can crush. Significant reduction in bioavailability and efficacy when administered with enteral nutrition. Pause the diet 1-hour before administration. Dilute with 10 mL of distilled water. |
| Cefalexin (500 mg e 1g, tablet coated) | Medicine has coating and excipients that can clog the tube when macerated. Alternative: Cephalexin Suspension. |
| Cefuroxime (250 mg and 500 mg, tablet coated) | It should not be macerated, according to the manufacturer's instructions. Alternative: Cefuroxime Suspension |
| Ciprofloxacin (500 mg tablet) | Simultaneous administration of the enteral diet may lead to decreased drug absorption. It is recommended to pause the enteral diet 1-hour before and 1-hour after administration. Bioavailability can range from 31%‒82% when co-administered with continuous EN. |
| Clarithromycin (500 mg, tablet coated) | There are no studies on efficacy, safety and pharmacokinetics. Alternative: Clarithromycin Suspension |
| Clindamycin (300 mg, cap) | It must not be broken or crushed. Open the capsule and disperse in 10 mL of water. |
| Doxycycline (100 mg tablet) | It can be shredded. Simultaneous administration with enteral diet may lead to reduced drug absorption. It is recommended to pause the enteral diet 1-hour before and after administration. |
| Fluconazole (150 mg, tablet) | It cannot shred. Open the capsule and wait for the granules to dissolve in 20 mL of water. Administer after complete solubilization. |
| Levofloxacin (500 mg, tablet coated) | Simultaneous administration of the enteral diet can lead to a decrease in drug absorption by up to 25% due to complexation with Ca, Zn, Mg and Al ions. It is recommended to pause the enteral diet 1-hour before and after. Crush the tablet, dilute in 10 mL of water. |
| Linezolid (600 mg, tablet coated) | Medicine has coating and excipients that can clog the tube when macerated. |
| Metronidazole (250 mg, tablet) | You can crush the tablet, dilute it in 10 mL of water, pause the diet 1-hour before and after washing the tube. |
| Moxifloxacin (400 mg, tablet coated) |  |
| Nitrofurantoin (100 mg, cap) | There are no studies on efficacy, safety and pharmacokinetics. Alternative: Nitrofurantoin Suspension. |
| Pyrimethamine (25 mg, tablet) | It can be shredded. Simultaneously administer enteral nutrition. |
| Sulfadiazine (500 mg, tablet) | The tablet can be crushed, dissolved in water to prevent crystalluria and administered immediately. |
| Sulphametoxazole + trimethoprim 400 mg + 80 mg, tablet ou 200 mg + 40 mg/ 5 mL, solution | The tablet has Active ingredient and excipients that when macerated can cause tube obstruction. Alternative: Sulfamethoxazole + Trimethoprim Suspension. |

Micromedex^®^ Healthcare Series [Internet database]. Greenwood Village, Colo: Thomson Healthcare.

ISMP, Brazil. Preparation and administration of medicines via enteral tube or ostomies. Available in http://www.ismp-brasil.org/site/wp-content/uploads/2016/03/Boletim-sondas.pdf.

GIMENES, F. R. E.; ANACLETO, T. A. (ORG. Preparation and administration of medicines via enteral tube or ISMP Brazil ostomies ‒ Institute for Safe Practices in the Use of Medicines. 2015.

Recommendations for medication administration via probe ‒ Pharmaceutical Dispensing Unit ‒ HU-UFGD/Ebserh, 2017. 17 p.

White, Rebecca, and Vicky Bradnam. Handbook of drug administration via enteral feeding tubes. Pharmaceutical Press, 2015.

Potter, Patricia. Nursing fundamentals. Elsevier Brazil, 2014.

Lima G; Negrini N.M.M. Pharmaceutical assistance in the administration of drugs via probe: choice of the appropriate pharmaceutical form. Einstein. 2009; 7(1 Pt 1):9-17.

**Table S3** Culture results by site of infections.

| **Site** | **Micro-organism** | **Number** |
| --- | --- | --- |
| Blood | *Enterobacter* spp | 1 |
|  | MRSA | 5 |
| Respiratory | *E. coli* | 1 |
|  | MSSA | 18 |
|  | MRSA | 10 |
|  | *Klebsiella* spp. | 7 |
|  | *A. baumannii* (carba-R) | 3 |
|  | *P. aeruginosa* | 3 |
|  | *Moraxella catarrhalis* | 1 |
|  | *Enterobacter* spp. | 2 |
|  | *Burkholderia cepacia +* MRSA | 1 |
|  | *E. coli* | 3 |
|  | *Klebsiella* spp. + MSSA | 2 |
|  | *E. coli +* MSSA | 1 |
|  | *Enterobacter* spp. + MSSA | 1 |
|  | *E. coli* | 1 |
|  | *Klebsiella* spp. + *Serratia* spp. | 1 |
|  | *Staphylococcus* spp. (non-aureus) | 2 |
|  | *Serratia* spp. | 1 |
|  | *Proteus* spp. | 1 |
|  | *Streptococcus* spp. | 2 |
|  | *Acinetobacter* spp. (carba-R) + *Enterobacter* spp. | 1 |
|  | *Enterobacter* spp. + *Klebsiella* spp. | 1 |
|  | *Pseudomonas* spp. + *E. coli* (ESBL) | 1 |
| Soft-Tissue | *Streptococcus spp.* | 1 |
|  | MRSA | 1 |
|  | *Klebsiella* spp. (ESBL) | 1 |
|  | Enterobacter spp. + Enterococcus spp. | 1 |
|  | *Leclercia* spp. + *Enterococcus* spp. | 1 |
|  | *Citrobacter* spp. | 1 |
| Urinary | *E. coli* | 2 |
|  | *Klebsiella* spp. | 1 |
|  | *Proteus* spp. | 1 |
| Others | *Staphylococcus* spp. (non-aureus) | 2 |

MRSA, Meticillin-Resistant *S. Aureus*; MSSA, Meticillin-Resistant *S. Aureus*; ESBL, Extended-Spectrum Beta-Lactamase; carba-R, Carbapenem-Resistant.
